# A Putative Plasma Membrane Na^+^/H^+^ Antiporter GmSOS1 Is Critical for Salt Stress Tolerance in *Glycine max*

**DOI:** 10.3389/fpls.2022.870695

**Published:** 2022-05-16

**Authors:** Minghui Zhang, Junfeng Cao, Tianxu Zhang, Tao Xu, Liyuan Yang, Xiaoyuan Li, Fengdan Ji, Yingxue Gao, Shahid Ali, Qingzhu Zhang, Jianhua Zhu, Linan Xie

**Affiliations:** ^1^Key Laboratory of Saline-Alkali Vegetation Ecology Restoration, Ministry of Education, College of Life Science, Northeast Forestry University, Harbin, China; ^2^State Key Laboratory of Tree Genetics and Breeding, Northeast Forestry University, Harbin, China; ^3^College of Life Science, Northeast Forestry University, Harbin, China; ^4^The Editorial Board of Journal of Forestry Research, Northeast Forestry University, Harbin, China; ^5^Laboratory Department, Qitaihe Center for Disease Control and Prevention, Qitaihe, China; ^6^Shanghai Center for Plant Stress Biology and Center for Excellence in Molecular Plant Sciences, Chinese Academy of Sciences, Shanghai, China; ^7^Department of Plant Science and Landscape Architecture, University of Maryland, College Park, College Park, MD, United States

**Keywords:** GmSOS1, soybean, Na^+^ efflux, salt tolerance, breeding

## Abstract

Soybean (*Glycine max*) is a staple crop and a major source of vegetable protein and vegetable oil. The growth of soybean is dramatically inhibited by salt stress, especially by the excessive toxic Na^+^. Salt Overly Sensitive 1 (SOS1) is the only extensively characterized Na^+^ efflux transporter in multiple plant species so far. However, the role of GmSOS1 in soybean salt stress responses remains unclear. Herein, we created three *gmsos1* mutants using the CRISPR-Cas9 system in soybean. We found a significant accumulation of Na^+^ in the roots of the *gmsos1* mutants, resulting in the imbalance of Na^+^ and K^+^, which links to impaired Na^+^ efflux and increased K^+^ efflux in the roots of the *gmsos1* mutants under salt stress. Compared to the wild type, our RNA-seq analysis revealed that the roots of the *gmsos1-1* showed preferential up and downregulation of ion transporters under salt stress, supporting impaired stress detection or an inability to develop a comprehensive response to salinity in the *gmsos1* mutants. Our findings indicate that the plasma membrane Na^+^/H^+^ exchanger GmSOS1 plays a critical role in soybean salt tolerance by maintaining Na^+^ homeostasis and provides evidence for molecular breeding to improve salt tolerance in soybean and other crops.

## Introduction

Soybean (*Glycine max*) is an important economic crop for livestock feed, human consumption, and industrial production because it is a major source for protein processing and vegetable oils extraction (Graham and Vance, 2003). Growing soybean cultivars on infertile soil, such as saline and drought areas, is harmful to ensuring staple crop production and enhancing overall food output. Seed germination in soybean is vulnerable to salt stress, and the yield of soybean is substantially decreased if soil salinity reaches 5 deciSiemens per meter (dS/m; a unit of electrical conductivity). Soybean is classified as a moderately salt-tolerant plant (Phang et al., 2008). Salt tolerance is an important trait in soybean breeding, and having a comprehensive understanding of the molecular basis of salt tolerance is important for crop breeding.

Under salt stress, ion homeostasis is maintained by adjusting the acquisition and distribution of K^+^ and Na^+^ in plants (Wu et al., 2018; Isayenkov and Maathuis, 2019; Rubio et al., 2019). Excess amounts of Na^+^ are supplied to roots, the main toxic ion of salinity, leading to accumulation in the photosynthetic tissues, ionic imbalance, cellular toxicity, metabolic disturbance, and reduced productivity (Hasegawa et al., 2000; Munns and Tester, 2008). Plants possess a series of pathways to minimize the toxicity of Na^+^, including limiting Na^+^ absorption, enhancing Na^+^ exclusion, altering cellular ionic balance (especially Na^+^/K^+^ ratio), and dispersing Na^+^ in leaves (Zhu, 2002; Zhu et al., 2016; Ishikawa and Shabala, 2019).

The membrane transporters are involved in Na^+^ and K^+^ influx and efflux processes and control the homeostasis of Na^+^ and K^+^. The monovalent cation/proton antiporter (CPA) family is one key group of ion transporters. The CPA family can be divided into two subfamilies: CPA1, which consists of Na^+^/H^+^ exchangers (NHXs), and CPA2, which encompasses K^+^ efflux antiporters (KEAs) and cation/H^+^ exchangers (CHXs) (Aviv, 1993; Chanroj et al., 2012; Ye et al., 2013). Ion transporters are critical for ion homeostasis under salt stress, and they often function in a coordinated manner with other components in the salt stress response pathways. Protein kinases, such as calcineurin B-like (CBL)-interacting protein kinases (CIPKs) and calcium-dependent protein kinases (CDPKs), have been found to respond to salt stress and participate in the regulation of plant salt stress tolerance (Weinl and Kudla, 2009; Boudsocq and Sheen, 2013).

In Arabidopsis, the Na^+^/H^+^ antiporter SOS1/Na^+^-H^+^ exchanger 7 (NHX7), the serine/threonine protein kinase SOS2/CIPK24, and the calcium-binding protein SOS3/CBL4 compose the minimal functional module in the Salt-Overly-Sensitive (SOS) signaling pathway (Martínez-Atienza et al., 2007; Quintero et al., 2011). SOS1 works as a homodimer, with each monomer having 12 transmembrane domains at its N-terminal region and a long C-terminal region containing a cytosolic domain, a cyclic nucleotide (cNMP)-binding domain, and an auto-inhibitory domain (Núñez-Ramírez et al., 2012). Under normal conditions in the resting state of SOS1, the C-terminal auto-inhibitory domain interacts with the adjacent activation domain containing a putative cNMP-binding motif, which is essential for SOS1 function (Shi et al., 2000). The SOS1 is regulated by cyclic nucleotides, particularly, signaling molecules in plant’s response to salt and osmotic stress that drive environmental adaptability (Shabala et al., 2015; Świeżawska et al., 2018). The Ca^2+^-dependent SOS2 – SOS3 protein kinase complex phosphorylates a serine residue at the position of 1,138 in the SOS1 auto-inhibitory domain, releasing SOS1 from auto-inhibition and activating SOS1 during salt stress (Quintero et al., 2011).

The physiological roles of the relevant *SOS1* genes have been widely investigated in plants in addition to *Arabidopsis thaliana*, including glycophyte, such as *Oryza sativa* (Razzaque et al., 2014), *Solanum lycopersicum* (Wang et al., 2021), *Chrysanthemum morifolium* (Gao et al., 2016), and *Gossypium hirsutum* (Chen et al., 2017), and halophytes, such as *Thellungiella salsuginea* (Oh et al., 2009), *Chenopodium quinoa* (Maughan et al., 2009), *Salicornia brachiata* (Yadav et al., 2012), and *Sesuvium portulacastrum* (Zhou et al., 2018). SOS1 exploits the H^+^ gradient to exclude Na^+^ from the cytosol by transporting Na^+^ into the extracellular space (Shi et al., 2000, 2002, 2003). A study on Oryza sativa Na^+^/H^+^ exchanger SOS1 further supports the role of SOS1 in exporting Na^+^ from the xylem parenchyma cells into the xylem vessels and thus promoting Na^+^ accumulation in the shoot for osmotic adjustment (Mahi et al., 2019). *GmSOS1* from the domesticated soybean (*Glycine max*), its wild relative (*Glycine soja*), and both their hybrids conferred high salt tolerance in transgenic Arabidopsis and yeast mutants (Nie et al., 2015; Zhao et al., 2017). However, its role in soybean plants under salt stress has not yet been investigated.

Here, we report that the loss-of-function mutants of the soybean GmSOS1 created with the CRISPR/Cas9 approach display exceptional increased salt sensitivity that correlates with excessive Na^+^ intake and accumulation in roots and leaves. Under salt stress, we observed a dramatic reduction of K^+^ contents in roots of the *gmsos1* mutant plants, while the salt-treated *gmsos1* plants accumulated similar levels of K^+^ in leaves as the wild-type plants. The ratio of Na^+^/K^+^ is much higher in the *gmsos1* mutants than in the wild-type plants under salt stress. Furthermore, the *gmsos1* mutations decrease the ability of Na^+^ efflux in the root meristem zone. Gene expression profiling with RNA-seq analysis revealed that many genes are differentially expressed in the *gmsos1* mutant plants under salt stress, and these genes encode proteins with diverse functions in cellular processes, including ion transport and response to abiotic stress. Our results demonstrate that SOS1 function is conserved in soybean and that the GmSOS1 is critical for salt tolerance in soybean.

## Materials and Methods

### Plant Materials and Salt Treatment

Seven soybean cultivars, namely, Williams 82 (W82), Jack, HeNong 85 (HN85), DongSheng 7 (DS7), HeiHe (HH43), DongNong 50 (DN50), and HeFeng 55 (HF55) were used in this study. All soybean materials used in this study were obtained from the Shanghai Center for Plant Stress Biology (CAS), Northeast Forestry University (NEFU), and Northeast Agricultural University (NEAU). Moderate size, disease-free, and intact seeds were planted in soil (native soil to vermiculite 2:1 ratio) at a temperature of 25°C under a 13 h light/11 h dark photoperiod for maturity. When cotyledons were fully grown (VE stage), seedlings were transferred to a hydroponic culture condition with a modified one-half-strength Hoagland nutrient solution [2.4 mM KNO_3_, 1.6 mM Ca(NO_3_)_2_, 0.2 mM KH_2_PO_4_, 0.8 mM MgSO_4_, 0.18 mM FeSO_4_, 0.1 mM Na_2_EDTA, 4.5 μM MnCl_2_, 23 μM H_3_BO_3_, 0.3 μM CuSO_4_, 1.5 μM ZnCl_2_, and 0.1 μM Na_2_MoO_4_] as previously described (López-Ráez et al., 2008) in a growth chamber for physiological measurements and assays.

The seedlings with uniform growth were selected and transferred to a culture box (a hard plastic rectangle-shaped container with black color to restrict growth of algae to house the one-half-strength Hoagland solution; see demonstration in Supplementary Figure 1) with varying concentrations of NaCl (0–200 mM NaCl) for salt stress treatments when the unifoliate leaves were fully expanded (VC stage). When the second trifoliolate leaf (V2 stage) in normal condition was fully opened, survival rate, plant height, fresh weight, and dry weight were recorded. At least 12 plants per genotype were used for salt tolerance analysis.

### Measurement of Na^+^ and K^+^ Contents

To determine Na^+^ and K^+^ contents, the collected plant samples (at approximately the V2 stage) were rinsed three times with sterile water to remove any impurities. The dry weight of the samples was determined after dehydration in an oven at 65°C for 5 days and then ground to fine powder. The powder was placed in a 50 ml conical bottle to be digested with appropriate volume of the mixed acid (nitric acid: hydrogen peroxide = 4:1) overnight. The sample solution was digested in an electric digestion system until all samples were clear and transparent. Deionized water was added to the final volume of 50 ml. The Na^+^ and K^+^ contents were measured by an inductivity coupled plasma mass spectrometry (ICP-MS; NexION 350D; PerkinElmer, Shelton, CT, United States) coupled to an Apex desolation system and an SC-4 DX autosampler (Elemental Scientific Inc., Omaha, NE, United States).

### Measurements of Net Na^+^ and K^+^ Fluxes

Net flux of Na^+^ was measured with the Non-invasive Microtest Technique (NMT, YoungerUSA, LLC, Amherst, MA, United States) and influxes software. For Na^+^ or K^+^ flux, seedlings at the VC stage were incubated in Hoagland medium supplemented with 160 mM NaCl for 24 h. Prior to the flux measurement, 10–20 mm lateral roots cut from the seedling were flushed with distilled water and soaked in the measuring solution [Na^+^: 0.1 mM CaCl_2_, 0.1 mM KCl, 0.5 mM NaCl, and 0.3 mM MES, adjusted pH to 6.0 with 1 M Tris-HCl (pH 8.8); K^+^: 0.1 mM KCl, 0.1 mM CaCl_2_, 0.1 mM MgCl_2_, 0.5 mM NaCl, 0.3 mM MES, and 0.2 mM Na_2_SO_4_, adjusted pH to 6.0 with 1 M Tris-HCl (pH 8.8)] for 10 min. Pre-pulled and salinized microsensors (4.5 ± 0.5 μm, XY- CGQ -01) were filled with a backfilling solution (Na^+^: 250 mM NaCl; K^+^: 100 mM KCl, pH 7.0) to a length of nearly 1.0 cm. Then, 50 μm columns of selective liquid ion-exchange cocktails (LIXs, Na^+^: XY-SJ-Na; H^+^: XY-SJ-H. Younger, United States) were filled from the tip. The microsensor was calibrated with 0.5 or 5 mM NaCl in calibration liquid (0.1 mM CaCl_2_, 0.1 mM KCl, 0.3 mM MES, pH 6.0) for measuring Na^+^ or K^+^ flux.

Ion fluxes were measured in the meristem zone of approximately 300 μm from the root tip. All the measurement results were exported using the JCal V3.3 (a free MS Excel spreadsheet^1^), and the consumables were provided by Xuyue (Beijing, China).

### Quantitative Reverse Transcription PCR Analysis

Total RNA was extracted from different tissues with the Trizol Reagent (Invitrogen, Waltham, MA, United States). Reverse transcription was performed using a PrimeScript™ RT reagent kit with gDNA Eraser (Perfect Real-time) reverse transcription Kit (TaKaRa, Kusatsu, Shiga, Japan). All quantitative real-time PCR analyses were performed using LightCycler 480 SYBR Green I Master (Roche, Basel, Switzerland). The transcript level of specific genes was measured using the cycle threshold (Ct) 2^–ΔΔ*Ct*^ method. The *GmTubulin A* gene was used as a reference gene (Klink et al., 2005; Wang et al., 2016). Primer sequences are listed in Supplementary Table 6.

### sgRNA Design and CRISPR/Cas9 Vector Construction

The single guide RNA (sgRNA) sequence (SG2, Supplementary Table 6) was designed based on the sequence of soybean *GmSOS1* gene (*GLYMA.08G092000.1*) downloaded from NCBI^2^. The U6 promoter from soybean and 35S promoter from cauliflower mosaic virus were used to drive sgRNA and *Cas9* gene, respectively. A *bar* gene conferring resistance to glufosinate in the plasmid is used as the marker for selecting the transgenic plants. Both forward and reverse primers of sgRNAs were denatured before annealing reaction (slow cool-down process from 95 to 16°C at 0.1°C/s). Two microliters of the annealed product were used to ligate to the pCBSG04 vector at 25°C for 30 min, and the reaction system includes the following components: 1 μl 10× Buffer (New England Biolabs, Ipswich, MA, United States), 1 μl Bsal-digested pCBSG04 plasmid, 2 μl Annealed product (1/100), 0.5 μl T_4_ Ligase (NEB), 0.1 μl T_4_ PNK (NEB), and 5.4 μl ddH_2_O.

### Plant Transformation and Screening for Mutated Plants

Two soybean cultivars, W82 and Jack, were utilized in the Agrobacterium-mediated tissue culture-based transformation. The transformation by *Agrobacterium tumefaciens* EHA105 (harboring the recombinant vector pCBSG04-GmU6: sgRNA-35S: Cas9) in soybean was previously described (Paz et al., 2004). All positive lines in T_0_ and T_1_ generations were confirmed by genotyping. After screening by 8 mg/L glufosinate, Genomic DNA was extracted by hexadecyltrimethylammonium bromide (CTAB) method from the glufosinate-resistant plants, and genomic fragments corresponding to sgRNA and Cas9 (primer: SOS1-F and SOS1-R, Supplementary Table 6) were amplified. The Cleaved *Amplified* Polymorphism Sequences (CAPS) marker was used to verify all the *gmsos1* mutants.

### Statistical Analysis

We used the R package and Excel to analyze the data in this study. Unless otherwise indicated, the Student’s *t*-test was used to compare the means at the level of *p* < 0.05 to find significant differences.

### Sequence Analysis of GmSOS1

We used MEGA X to construct an evolutionary tree with full-length GmSOS1 and SOS1 proteins from another 21 plant species as described (Kumar et al., 2018). Multiple sequence alignment of GmSOS1 with AtSOS1 was performed with the DNAMAN software (version 5.0; Lynnon BioSoft, Canada).

### RNA-Seq Analysis

Total RNA was extracted from different tissues of the wild type and *gmsos1-1* plants with Trizol reagent (Invitrogen, Waltham, MA, United States). The concentration and quality of each RNA sample were determined on an Agilent 2100 Bioanalyzer (Agilent Technologies, Waldbronn, Germany), and 3 μg of total RNA from each sample were used for RNA-seq library construction. There are three biological replicates per genotype. Sequencing of the RNA-seq libraries was performed using the Illumina HiSeq 2500 platform (Illumina, San Diego, CA, United States). Approximately 4.0 Gb of clean data were generated per sample. We processed and analyzed the RNA-seq data following procedures as previously described (Sahraeian et al., 2017). Briefly, all low-quality (<Q30) paired-end reads were excluded, and the reads were trimmed using trimmomatic (vision 0.38). The trimmed reads were mapped to the soybean reference genome (Phytozome v2.1) using hisat2, and the stringtie was used to assemble expressed transcripts. We subsequently used DESeq2 to identify the differentially expressed genes (DEGs) with | log2(Fold change)| > 1 and adjusted the false discovery rate (FDR) < 0.01. AgriGo and R were used to perform the Gene Ontology (GO) analyses of the DEGs (Du et al., 2010), with FDR < 0.05 as a cutoff for significantly enriched GO terms.

## Results

### Williams 82 Shows Moderate Tolerance to Salt Stress

**FIGURE 1:**
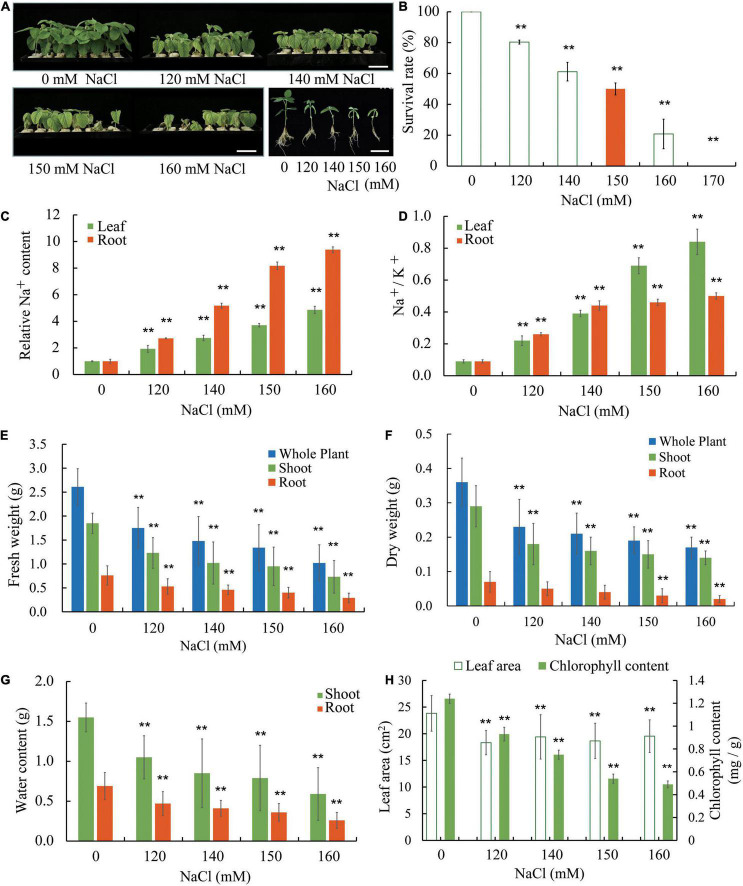
Salt sensitivity of Williams 82 (W82) plants. **(A)** Growth of seedlings in liquid growth medium containing 0, 120, 140, 150, or 160 mM NaCl. Bar = 6 cm. **(B)** The survival rates of plants as shown in **(A)** and data for plants treated with 170 mM NaCl are also provided. Survival is defined as the ability of shoot apical meristem to initiate new trifoliate leaves after the salt stress treatment. **(C)** The relative Na^+^ content in roots and unifoliate leaves of plants as shown in **(A)**. **(D)** The Na^+^/K^+^ ratios in roots and unifoliate leaves from plants as shown in **(A)**. **(E)** The fresh weight of whole plants, shoots, and roots of plants as shown in **(A)**. **(F)** The dry weight of whole plants, shoots, and roots of plants as shown in **(A)**. **(G)** The relative water content of shoots and roots of plants as shown in **(A)**. **(H)** The chlorophyll content and the area unifoliate leaves of plants as shown in **(A)**. The data are means ± SD [*n* = 3 (there are at least 12 plants in each biological replicate)]. Significant differences in mean values relative to the mean value of unstressed plants are indicated by Student’s *t*-tests (**p < 0.01).

Williams 82 is the soybean cultivar which was sequenced to produce the reference genome sequence. To gain some insight on how soybean plants respond to salt stress, we used W82 in our experiment where in young seedlings were grown in a hydroponic condition. Seedlings at the vegetative cotyledon (VC) stage were transferred to Hoagland solutions and supplemented with various concentrations of NaCl (0, 120, 140, 150, and 160 mM). The seedlings were allowed to grow for an additional 8 days, a growth period during which seedlings without salt stress would transit from the VC stage to the second trifoliate (V2) stage (Figure 1A and Supplementary Figure 1). Compared with seedlings under control conditions, seedlings show reduced growth and development at low levels of salt stress and display severe growth retardation accompanied by wilting and, at times, death at high levels of salt stress (Figures 1A,B,E–H and Supplementary Figure 1). We used the ability of shoot apical meristem of the salt-treated seedlings to initiate new trifoliate leaves as a criterion to judge whether a seedling survives salt stress. It is obvious to notice that salt stress significantly reduces the survival rate of soybean seedlings (Figure 1B). It appears that approximately 50% of the seedlings can survive under 150 mM NaCl. Increasing amount of Na^+^ accumulated in roots and leaves of salt-treated Williams 82 seedlings and roots had substantially higher levels of Na^+^ than the leaves (Figure 1C). We also determined the ratio of Na^+^ and K^+^ (Na^+^/K^+^) in the W82 seedlings and found that Na^+^/K^+^ increases as the NaCl concentration rises. The Na^+^/K^+^ values are higher in leaves than roots when the seedlings are treated with 150 and 160 mM NaCl (Figure 1D). These results suggest that there is an internal mechanism to control Na^+^ ion distribution from roots to shoot tissues, such as leaves, to prevent the overaccumulation of this toxic ion in the shoots.

### Degree of Salt Tolerance Varies Amongst Soybean Cultivars

**FIGURE 2:**
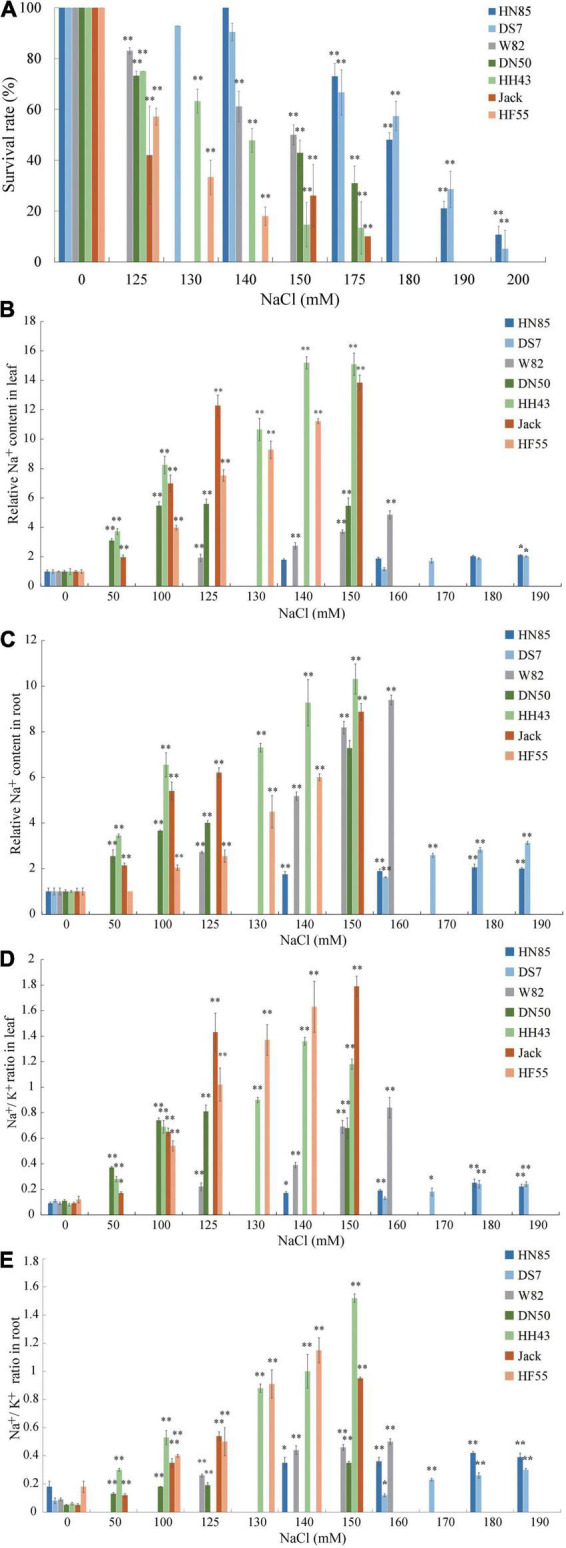
Salt sensitivity assay of six soybean cultivars. **(A)** Survival rates of six soybean cultivars subjected to treatment with 0, 125, 130, 140, 150, 175, 180, 190, or 200 mM NaCl. The six soybean cultivars are Henong 85 (HN85), Dongsheng 7 (DS7), Dongnong 50 (DN50), Heihe 43 (HH43), Jack (Jack), and Hefeng 55 (HF55). **(B)** The Na^+^ contents in unifoliate leaves of the six soybean cultivars. **(C)** The Na^+^ contents in roots of the six soybean cultivars. **(D)** The change of Na^+^/K^+^ ratios in unifoliate leaves of the six soybean cultivars. **(E)** The change of Na^+^/K^+^ ratios in roots of the six soybean cultivars. Data are means ± SD [*n* = 3 (there are at least 12 plants in each biological replicate)]. Significant differences in mean values relative to the mean value of unstressed plants are indicated by Student’s *t*-tests (*p < 0.05, **p < 0.01).

To further evaluate whether there are variations in salt tolerance in different soybean germplasms, six randomly selected cultivars from Northeast China: HeNong 85 (HN85), DongSheng 7 (DS7), DongNong 50 (DN50), HeiHe 43 (HH43), HeFeng 55 (HF55), and Jack are used in the hydroponic evaluation system described above (W82 is used as a control) (Figure 2). First, the survival rates of all cultivars were determined at varying concentrations of NaCl treatment (50 mM to 200 mM) to define the salt concentration at which each cultivar’s survival rate is around 50%. These were referred to as half survival concentrations (HSCs). The HSC is 180 mM NaCl for HN85 and DS7, which is the highest among the seven cultivars, the HSC for Jack and HF55 is 125 mM NaCl, which is the lowest among the cultivars tested, and the HSC for DN50 and HH43 is 150 mM NaCl (the same as W82) (Figure 2A). The HSC values also indicate the salt tolerance levels. This means that the HN85 and DS7 cultivars are the most salt-tolerant among the cultivars, the HN43, DN50, and W82 cultivars are moderately salt-tolerant, and the Jack and HF55 cultivars are least salt-tolerant among the seven cultivars.

To validate the salt stress responses of the six cultivars, the unifoliolate leaves and roots at the first trifoliate (V1) stage from salt-treated seedlings were separately collected for quantitative evaluation of the Na^+^ and K^+^ contents. Although all cultivars’ relative Na^+^ contents in roots and leaves are increasing, it is noted that the slopes are moderate in salt-tolerant cultivars and steep in salt-sensitive cultivars (Figures 2B,C). Similar trends were found in the Na^+^/K^+^ values in unifoliate leaves (Figure 2D). In the roots, the salt-sensitive cultivars have higher values of Na^+^/K^+^ than the salt-tolerant cultivars, except for DS7 (Figure 2E).

### Generation of *gmsos1* Mutants by CRISPR/Cas9 Approach

We predict that GmSOS1 is important in salt tolerance in soybean. Database searches and sequence comparisons, such as phylogenetic analysis and conserved domain prediction, revealed that the NP_001244939.1 (encoded by a single-copy gene *GLYMA.08G092000.1*) protein from the W82 reference genome shows high sequence homology with the plasma membrane localized Na^+^/H^+^ Exchanger SOS1 family proteins from *A. thaliana* (NP_178307.2 encoded by *AT2G01980.1*) and *O. sativa* (AAW33875.1) (Supplementary Figures 2A,B). We subsequently named *GLYMA.08G092000.1* as *GmSOS1*. GmSOS1 is predicted to be a cell membrane protein by the Plant-mPloc program^3^. GmSOS1 has 12 transmembrane helices at its N-terminal portion (from amino acids 35--446) as predicted by the TMHMM program^4^, a typical structure of most plasma membrane localized ion transporter proteins.

**FIGURE 3:**
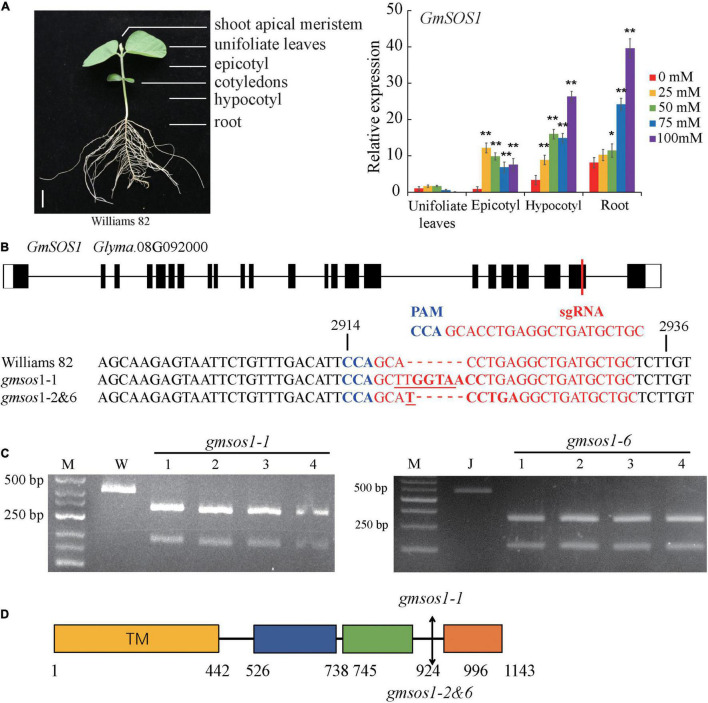
Generation of *gmsos1* mutants using CRISPR/Cas9 system. **(A)** The relative transcript levels of *GmSOS1* in different tissues as determined by qRT-PCR analysis. The RNA samples were from unifoliate leaf, root, epicotyl, and hypocotyl of Williams plants that are subjected to 0, 25, 50, 75, or 100 mM NaCl. Values are means ± SD (*n* = 3). Significant differences in mean values relative to the mean value of unstressed plants in each tissue are indicated by Student’s *t*-tests (*p < 0.05, **p < 0.01). **(B)** Generation of mutations in *GmSOS1* (Glyma.08G092000) by CRISPR/Cas9 using single-guide RNA (sgRNA). The sgRNA sequence is in red, and the protospacer adjacent motif (PAM) sequence is in blue. The coding sequence of *GmSOS1* that is complementary to the sgRNA and its flanking sequences are compared with the corresponding sequences in *gmsos1-1*, *gmsos1-2*, and *gmsos1-6* mutants. The sequence in bold in *gmsos1-1* is the *Bst*E II digestion site (GGTNACC) and the sequence in bold in *gmsos1-2* and *gmsos1-6* is a *Hpy*188 III digestion site (TCNNGA). The numbers (2,914 and 2,936) above the nucleotide sequences are relative to the start codon of GmSOS1. **(C)** Cleaved amplified polymorphic sequence (CAPS) markers were used to identify the mutations in the *gmsos1* mutants. PCR products amplified by specific primers from Williams (W) and the *gmsos1-1* plants were digested by *BstE* II (left panel). PCR products amplified by specific primers from Jack and the *gmsos1-6* plants were digested with *Hpy188* III (right panel). Primers used in this experiment were listed in Supplementary Table 1. M represents DNA ladder. **(D)** The mutations identified in the *gmsos1* mutants and potential effects on the GmSOS1 protein. Yellow box (from 1 to 442 aa), putative N-terminal transmembrane domain; blue box (from 526 to 738 aa), putative intracellular domain homologous to AtNHX8; green box (from 745 to 924 aa), putative C-terminal cyclic nucleotide (cNMP)-binding domain; orange box (from 996 to 1,143 aa), putative self-inhibition domain.

The expression patterns of *GmSOS1* in various tissues of W82 under salt stress were determined to understand the role of GmSOS1 in salt tolerance (Figure 3A). The expression of *GmNAC004* is responsive to salt stress, indicating that our salt stress treatments are effective (Supplementary Figure 2C). The expression levels of *GmSOS1* are the highest in the roots compared to the other tissues, such as leaves, epicotyls, and hypocotyls (Figure 3A). Intriguingly, the expression of *GmSOS1* in tissues above the cotyledon, such as the leaf and epicotyl, and tissues below cotyledon, such as the hypocotyl and root, showed distinct patterns when exposed to different levels of salt stress. Despite its modest expression level in control, *GmSOS1* is downregulated in leaf and epicotyl when the NaCl concentration rises, while the expression of *GmSOS1* in the hypocotyl and root increases as the levels of salt stress increases (Figure 3A). These results suggest that *GmSOS1* responds to salt stress and may help maintain a low sodium concentration in sensitive organs by contributing to Na^+^ efflux in the root and transportation from root to aboveground tissues. To determine whether GmSOS1 is critical for salt tolerance in soybean, we designed a sgRNA complementary to the nucleotide sequences whose amino acid residues are in the linker region of the autoinhibitory domain and cNMP-binding domain of GmSOS1 to create mutations in *GmSOS1* in the W82 and Jack backgrounds with the CRISPR/Cas9 approach (Figures 3B,D). We generated two mutations in *GmSOS1* that correspond to 3 mutants: *gmsos1-1* and *gmsos1-2* in the background of W82, and *gmsos1-6* in the background of Jack (Table 1). The *gmsos1* mutations were validated by PCR amplifications with primers containing CAPS markers, followed by restriction enzyme digestions (Figure 3C). The 6-bp insertion mutation of the *gmsos1-1* introduced a *Bst*EII recognition site (GGTNACC) so that the PCR fragment from the mutant can be digested to produce two fragments: the 344-bp longer fragment and the 169-bp shorter one. The 1-bp insertion mutation of the *gmsos1-2 and gmsos1-6* introduced a *Hpy*188III recognition site (TCNNGA) so that the PCR fragment from either the *gmsos1-2* or *gmsos1-6* plants can be digested to produce two fragments: the 344-bp longer fragment and the 169-bp shorter one. Seeds from the homozygous *gmsos1* mutants in the T_2_ or T_3_ generations are used in the subsequent experiments. The *gmsos1-1* has a 6-bp insertion, and this insertion would make a frameshift and produce a premature stop codon at the amino acid residue 975. The *gmsos1-2* and *gmsos1-6* have 1-bp insertion, and this insertion would make a frameshift and produce a premature stop codon at the amino acid residue 975. The *gmsos1-1*, *gmsos1-2*, or *gmsos1-6* mutations would produce a truncated polypeptide that is missing the last 169 amino acids from the C-terminal end of wild-type GmSOS1 (Figure 3D).

**TABLE 1:** List of the *gmsos1* mutants in this study.

Genotype	Indel	Deduced aa sequence around the mutation region	Background
Williams 82	Not applicable	968 LFDIPA _– –_ PEADAA	Williams 82
*gmsos1-1*	+6 bp (TTGGTA)	968 LFDIPAW*	Williams 82
*gmsos1-2*	+1 bp (T)	968 LFDIPAS*	Williams 82
*gmsos1-6*	+1 bp (T)	968 LFDIPAS*	Jack

*indicates a premature translation stop codon.

### The *gmsos1* Mutants Are Hypersensitive to Salt Stress

**FIGURE 4:**
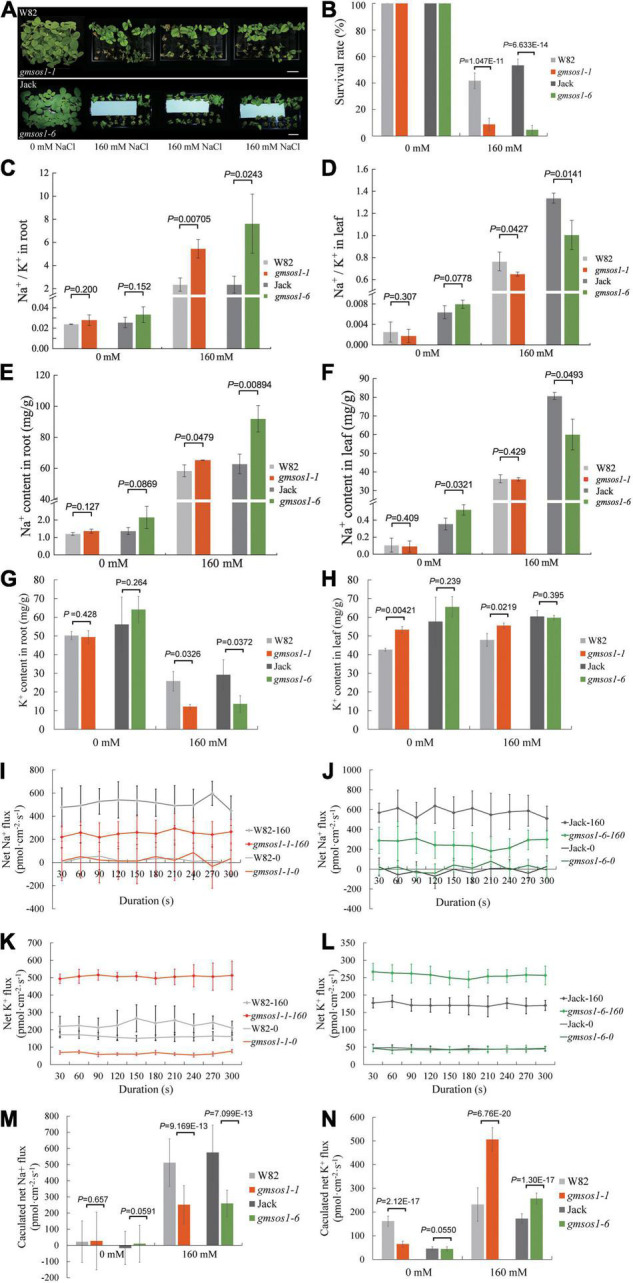
The *gmsos1* mutants are more sensitive to salt stress than wild-type plants. **(A)** Growth of the *gmsos1* mutants and wild type plants in liquid medium containing 0 or 160 mM NaCl. Bar = 6 cm. **(B)** Survival rates of the *gmsos1* mutants and wild-type plants as shown in **(A)**, the number of the plant is 12 in 3 repeated experiments. The Na^+^/K^+^ ratios in roots **(C)** and in unifoliate leaves **(D)**, Na^+^ content in roots **(E)**, Na^+^ content in unifoliate leaves **(F)**, K^+^ content in roots **(G)**, and K^+^ content in unifoliate leaves **(H)** of plants as shown in **(A)**. Net Na^+^ flux analysis at the meristem zones of wild type and *gmsos1-1* **(I)** and *gmsos1-6* **(J)** mutants. Net K^+^ flux analysis at the root meristem zones of wild type and *gmsos1-1* **(K)** and *gmsos1-6* **(L)** mutant. **(M)** Calculated net Na^+^ fluxes from Net Na^+^ flux analysis at the meristem zone of wild-type and mutant roots. **(N)** Calculated net K^+^ fluxes from Net K^+^ flux analysis at the meristem zone of wild types and mutants. Data are means ± SD [*n* = 3 in (**B**, there are 12 plants per biological replicate), 6 in **(C–N**, there are at least 12 plants per biological replicate)]. *p*-values from Student’s *t*-tests were added in **(B)–(H)**, **(M)**, and **(N)**.

We examined the effects of the *gmsos1-1* and *gmsos1-6* mutations on salt tolerance with the hydroponic system. Compared with their relevant wild-type plants, the *gmsos1-1* and *gmsos1-6* plants display severe retarded growth or, at times, death and much lower survival rates under salt stress, indicating that they are hypersensitive to salt stress (Figures 4A,B). The roots of the *gmsos1* mutants essentially accumulated the same amounts of Na^+^ as the roots of the wild-type seedlings under control conditions, but they accumulated substantially higher levels of Na^+^ under salt stress than the roots from the wild-type seedlings (Figure 4E). Meanwhile, the roots of the *gmsos1* mutants had similar amounts of K^+^ as the roots from the wild-type seedlings under control conditions, but they accumulated significantly lower levels of K^+^ under salt stress than the roots of the wild-type seedlings (Figure 4G). As a result, the roots of the *gmsos1* mutants had higher levels of Na^+^/K^+^ values than the roots of their wild-type seedlings under salt stress (Figure 4C). The unifoliate leaves of the *gmsos1-6* seedlings accumulated lower amounts of Na^+^ than the unifoliate leaves from the Jack seedlings under control and salt stress conditions, whereas the unifoliate leaves of the *gmsos1-1* seedlings had essentially similar levels of Na^+^ as the unifoliate leaves of the W82 seedlings under control and salt stress conditions (Figure 4F). The unifoliate leaves of the *gmsos1-1* seedlings accumulated higher amounts of K^+^ than the unifoliate leaves of the W82 seedlings under control and salt stress conditions, whereas the unifoliate leaves of the *gmsos1-6* seedlings had essentially similar levels of K^+^ as the unifoliate leaves of the Jack seedlings under control and salt stress conditions (Figure 4H). Because of the imbalanced accumulations of Na^+^ and K^+^, the unifoliate leaves of the *gmsos1* mutants had lower Na^+^/K^+^ values than the unifoliate leaves of their wild-type seedlings under salt stress. Meanwhile, the unifoliate leaves of the *gmsos1-6* seedlings had lower Na^+^/K^+^ value than the unifoliate leaves of the Jack seedlings under control conditions (Figure 4D). To examine in detail the *gmsos1* mutant plants’ responses to salt stress, net Na^+^ or K^+^ flux in the root meristem zones of the *gmsos1-1* and *gmsos1-6* seedlings at the VC stage that were subjected to 160 mM NaCl treatment for 24 h were measured using Non-invasive Micro-Test Technology (NMT) within a continuous period of 300 s to show the dynamics of the Na^+^ or K^+^ flow. The *gmsos1* mutants did not show any significant differences in net Na^+^ flux in the root meristem zones compared to their relevant wild-type seedlings under control conditions, but these mutants had slower net Na^+^ flux in the root meristem zones compared to their relevant wild-type seedlings under salt stress conditions (Figures 4I,J,M). The *gmsos1-1* seedlings had slower net K^+^ flux in the root meristem zone than the W82 seedlings under control conditions, but had higher net K^+^ flux in the root meristem zone than the W82 seedlings under salt stress condition (Figures 4K,N). In contrast, the *gmsos1-6* seedlings only had higher net K^+^ flux in the root meristem zone than the Jack seedlings under salt stress condition, while the mutant and the wild-type seedlings did not differ in net K^+^ flux in the root meristem zone under control conditions (Figures 4L,N). These results suggest that Na^+^ efflux in the *gmsos1* mutants may be impaired due to the loss-of-function of GmSOS1, and that K^+^ efflux is enhanced in the *gmsos1* mutants under salt stress.

### Transcriptome Analysis of the *gmsos1-1* Mutant

**FIGURE 5:**
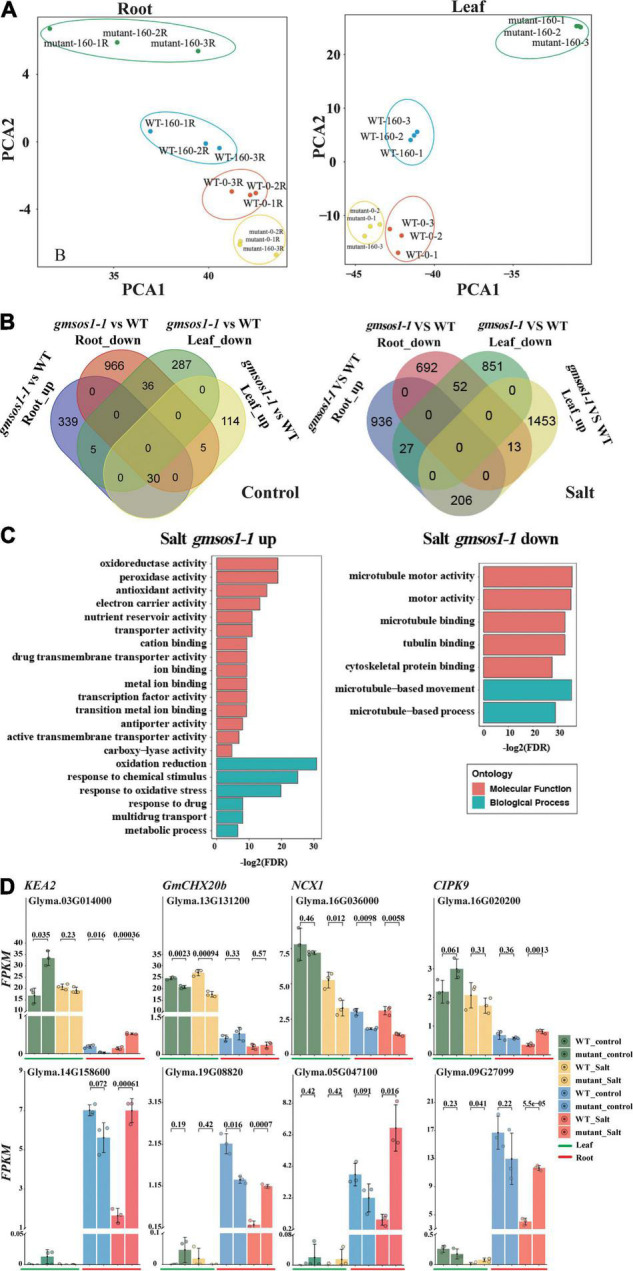
Summary of differentially expressed genes (DEGs) in the *gmsos1-1* mutant. **(A)** Principal component analysis of the 2,000 most significant DEGs in root and leaf of the *gmsos1-1* and wild-type plants (*n* = 3 biological replicates). **(B)** Four-way Venn diagram indicating the number of upregulated and downregulated genes in the unifoliate leaves and roots of *gmsos1-1* mutant under control conditions (left panel). Four-way Venn diagram indicating the number of upregulated and downregulated genes found in the comparison between the roots of *gmsos1-1* mutant and wild type under 160 mM NaCl treatment (right panel). **(C)** Gene-ontology analysis of the upregulated and downregulated genes in salted *gmsos1-1* mutant. **(D)** Fragments per kilobase million (FPKM) of significantly upregulated and downregulated DEGs found in unifoliate leaves and roots of *gmsos1-1* mutant and the wild-type W82 under 160 mM NaCl treatment. Error bars indicate standard deviation (*n* = 3). *P*-values are from Student’s *t*-tests.

To further examine the global influence of the *gmsos1-1* mutation on gene expression, we performed an RNA-seq analysis with roots and unifoliolate leaves from the W82 (WT) and *gmsos1-1* plants under normal and salt stress conditions. We identified DEGs in the roots and unifoliate leaves of the *gmsos1-1* under normal and salt stress conditions by the threshold of fold changes > 2 and adjusted FDR < 0.01 (Supplementary Tables 1, 2). Furthermore, principal component analysis (PCA) of the 2,000 most significant DEGs in the roots and unifoliate leaves of the wild-type and *gmsos1-1* plants graphically confirmed that the DEG analysis was reliable (Figure 5A). Under the control conditions, there are 1,007 downregulated and 374 upregulated genes in the roots of the *gmsos1-1*, while there are 328 downregulated genes and 149 upregulated genes in the unifoliate leaves of the *gmsos1-1* (Figure 5B and Supplementary Table 1). It seems that salt stress treatments led to a stronger effect on gene expression in the *gmsos1-1* plants. Under the salt treatment, there are 692 downregulated genes and 936 upregulated genes in the roots of the *gmsos1-1*, while there are 851 downregulated genes and 1,453 upregulated genes in the unifoliate leaves of the *gmsos1-1* (Figure 5B and Supplementary Table 2).

To evaluate the potential biological function of the DEGs in the *gmsos1-1* plants in response to salt treatment, GO analysis was performed using the AgriGO2 service (Du et al., 2010) for DEGs identified in the *gmsos1-1* (Figure 5C and Supplementary Table 3). The 760 salt-responsive upregulated *DEGs* were clustered into 30 substantially enriched GO categories, with six biological processes and 18 functional categories (Figure 5C and Supplementary Table 4). The 693 salt responsive downregulated *DEGs* were clustered into seven substantially enriched GO categories, two of which corresponded to biological processes and the other five to functional categories (Figure 5C and Supplementary Table 4). The *DEGs* encode proteins with diverse functions in cellular processes. The top GO categories of the upregulated *DEGs* include “oxidation-reduction” (GO:0055114, 90 genes; FDR of 5.40E-10), “response to oxidative stress” (GO:0006979, 20 genes; FDR of 1.10E-06), “cation binding” (GO:0043169, 91 genes; FDR of 0.0016), and “antiporter activity” (GO:0022804, 17 genes; FDR of 0.004) (Supplementary Table 3). The top enriched GO categories of the downregulated *DEGs* in the salt-treated roots of the *gmsos1-1* are microtubule network-related genes (GO:0007018, 20 genes; FDR of 2.10E-11) (Supplementary Table 2). There are 54 *DEGs* in the *gmsos1-1* that encode proteins related to ion homeostasis (Supplementary Table 5). We paid particular attention to the expression levels of selected DEG encoding proteins potentially involved in the ionic component of salt stress, primarily Na^+^ and K^+^ transporters in the root and leaf, respectively (Supplementary Table 5). In Arabidopsis, *KEA* genes are expected to encode K^+^/H^+^ antiporters. *GmKEA2* (*Glyma.03G014000*) is highly expressed in leaves, and it is upregulated in *gmsos1-1* under control conditions (Figure 5D and Supplementary Table 5). *GmKEA2* is expressed to a much lower level in the roots, and its level is greater in the *gmsos1-1* than in the wild type (Figure 5D and Supplementary Table 5). *GmCHX20b* (*Glyma.13G131200*) encodes a cation/H^+^ exchanger (Jia et al., 2021), and its expression in roots was significantly lower than in leaves under control and salt stress conditions. In addition, its level is reduced in the leaves of the *gmsos1-1* plants (Figure 5D and Supplementary Table 5). Furthermore, as a member of the Na^+^/Ca^2+^ exchanger (NCX) protein family, it plays important roles in cellular Ca^2+^ homeostasis (Singh et al., 2015). The expression of *GmNCX1* (*Glyma.16G036000*) in roots was significantly lower than in leaves under control and salt stress conditions, and is upregulated in the roots of the *gmsos1-1* plants (Figure 5D and Supplementary Table 5). *Glyma.16G020200* encodes the CBL-interacting protein kinase 9 (GmCIPK9). The expression of *GmCIPK9* in roots is lower than in leaves under control and salt stress conditions, and is downregulated in the unstressed roots and salted-treated leaves and roots of the *gmsos1-1* plants (Figure 5D and Supplementary Table 5). *Glyma.14G158600*, *Glyma.19G088200*, *Glyma.05G047100*, and *Glyma.09G270900* encode EF-hand (a diverse motif class consisting of 30 amino acids that fold into a helix-loop-helix structure; the loop between the helices comprised of ~12 conserved amino acids coordinates a calcium ion) domain-containing proteins and they have higher expression levels in the roots than in the leaves (Figure 5D and Supplementary Table 5). They are upregulated in the roots of the *gmsos1-1* plants under salt stress (Figure 5D and Supplementary Table 5).

## Discussion

Soybean is classified as a moderately salt-tolerant crop that displays a spectrum of salt-tolerance-related phenotypes (Phang et al., 2008). The salt-sensitive cultivars had a 37% decrease of yield than the salt-tolerant cultivars under saline conditions (Parker et al., 1983). At the seed germination and young seedling developmental stages, evaluation of soybean germplasms under salt stress employed cultivars of soybean from various regions, which showed diverse phenotypes that were sensitive, moderate, and tolerant under salt stress (Kan et al., 2016; Do et al., 2019). Efficient evaluation systems of soybean salt tolerance are lacking and could negatively impact the elucidation of molecular components in salt stress response pathways and breeding of salt-tolerant crops in the long term. In this study, we established a robust and quick salt-sensitivity screening for soybean cultivars. In addition, we primarily used the physiological parameters of seedling survival rate, accumulation of Na^+^ and K^+^ in the roots and leaves, and ratio of Na^+^/K^+^ in those two tissues.

There is a correlation between salt tolerance and Na^+^ accumulation and the Na^+^/K^+^ ratio (Wang et al., 2020). Comparative studies have shown that salinity-tolerant cultivars accumulate lower levels of Na^+^ in leaves and shoots compared with those in the salinity-sensitive cultivars. In addition, a lower Na^+^/K^+^ ratio is associated with salt tolerance (Golldack et al., 2003; Lee et al., 2003; Lin et al., 2004; Ren et al., 2005; Munns and Tester, 2008; Mahi et al., 2019; Wang et al., 2020). Hence, the quantitative measure of the Na^+^ and K^+^ concentrations in shoots and roots in salinized conditions has often been used as a trait in quantitative trait loci mapping, salt tolerance evaluation, and crop breeding programs (Hoang et al., 2016; Mahi et al., 2019). In this study, the contents of Na^+^ and K^+^ and the ratios of Na^+^/K^+^ in the roots are all significantly higher in the sensitive soybean cultivars and *gmsos1* mutants. In contrast, there is no substantial differences in the contents of Na^+^, K^+^, or the Na^+^/K^+^ ratios in leaves of the sensitive soybean cultivars and *gmsos1* mutants. One reason might be that *GmSOS1* is highly expressed in the roots (Figure 3A), which are the most sensitive organ for Na^+^ accumulation and flow.

The membrane bound ion transporters are responsible for Na^+^ absorption, exclusion, compartmentation, and redistribution. The salt-tolerant cultivars HN85 and DS7 had much lower levels of Na^+^ in the leaves and roots, along with smaller Na^+^/K^+^ ratios in the roots, indicating that genes involved in Na^+^ efflux at high NaCl concentrations may be significant in these salt-tolerant cultivars. The results suggest that the sodium flux transporters are responsible for maintaining the intracellular Na^+^ concentration at low levels. The Na^+^/H^+^ antiporter SOS1 is one of the most important components in plant salt tolerance that is known so far. *GmSOS1* is specifically expressed in roots and less expressed in leaves, epicotyls, and hypocotyls, and it is inducible by increasing salt stress levels. *GmSOS1* shows similar expression patterns with the *SOS1* genes from Arabidopsis, rice, and other plant species, which might contribute to the Na^+^ efflux in the roots to maintain low Na^+^ concentration in the sensitive organ.

In Arabidopsis, the C-terminal auto-inhibitory domain of SOS1 interacts with the adjacent activation domain to keep the resting status of basal activity without stress when the Ca^2+^-dependent SOS2-SOS3 protein kinase complex phosphorylates SOS1 and relieves SOS1 from auto-inhibition for an induced level upon salinity stress. The interaction of the auto-inhibitory domain with an activation domain is essential for the SOS1 activity (Quintero et al., 2011). There is evidence that a mutation, clustered at the C-terminal region between the activation and auto-inhibitory domains, causes salt tolerance which may hinder the activation and auto-inhibitory domain from interacting *in vivo*, thereby resulting in a constitutively active full-length SOS1 protein (Quintero et al., 2011). In this study, we designed a sgRNA that targets the nucleotide sequences corresponding to the linker of activation and auto-inhibitory domains, with an expectation to create point mutations in GmSOS1. In addition, to possibly create a constitutively active GmSOS1. We failed to identify a soybean plant carrying a constitutively active GmSOS1 in this study. Such a mutation may require successful precise base-pair editing technology, which is not currently well-developed. We were able to recover three mutants in GmSOS1 in this study: *gmsos1-1*, *gmsos1-2*, and *gmsos1-6*. These mutants have premature stop codons and created C-terminal deletions. The truncated GmSOS1 proteins from the *gmsos1-1*, *gmsos1-2*, and *gmsos1-6* mutants might lack the target site (i.e., an amino acid residual equivalent to the serine 1,138 of AtSOS1) to be phosphorylated to activate GmSOS1 protein activity (Quintero et al., 2011). The roots of these mutants that lose SOS1 activity display decreased Na^+^ flux and increased accumulations of Na^+^, leading to hypersensitive responses to salt stress. The *gmsos1* mutants have similar salt hypersensitive phenotypes as the *sos1* mutants from other plant species, such as Arabidopsis and rice, suggesting that SOS1 function is conserved in eudicot and monocot angiosperms.

High salinity imposes water stress, which is the osmotic effect of the saline solution outside the cell, and an ionic imbalance, resulting primarily from the accumulation of Na^+^ and the loss of K^+^ (Munns and Tester, 2008). Roots are the main defensive barrier against salinity and is the organ that first senses this soil-derived stress (Munns and Tester, 2008). Leaves are the most sensitive organs to sense salinity. Hence, we performed whole-transcriptome sequencing (RNA-seq) analysis of roots and unifoliolate leaves from the W82 and *gmsos1-1* plants under the normal and salt stress conditions to further investigate the global effect of the *gmsos1-1* mutation on gene expression. We observed that a large number of genes whose expression was altered in the *gmsos1-1* mutant plants. These DEGs encode proteins with diverse functions in nearly all cellular processes. There are over 50 DEGs in the roots of the salt-treated *gmsos1-1*, and these genes encodes proteins involved in ion homeostasis. Among the salt stress responses, ion homeostasis is essential for plant growth and development under environmental stresses. The monovalent CPA family members have evolved to accomplish this. *AtKEA*s encode K^+^/H^+^ antiporters in Arabidopsis and play important roles in facilitating K^+^ homeostasis and osmotic adjustment (Zheng et al., 2013). The expression of the *AtKEA2* in *atsos1* mutants shows a higher expression level under 160 mM NaCl (Zheng et al., 2013). In this study, *GmKEA2* was upregulated in roots of the *gmsos1-1* under salt stress, which shows a similar expression pattern as in the Arabidopsis *atsos1* mutant plants. Our data demonstrated that the sensitivity of soybean seedlings to salt is strongly associated with the decreasing K^+^ contents in the roots and the accumulation of Na^+^ in the roots. The upregulation of *GmKEA2* in the salinized *gmsos1-1* roots might lead to the efflux of potassium ions in the roots and hypersensitive phenotype in the *gmsos1-1* mutant plants. Studies on *GmCHX20a* and *GmCHX1* indicate that cation/H^+^-exchangers (CHXs) perform diverse functions in plants to cope with salt stress. *Cation/H*^+^*-exchanger1* (*GmCHX1*) is the causal gene in the major salt tolerance quantitative trait locus (QTL) in soybean that reduces the accumulation of Na^+^ in leaves (Guan et al., 2014; Qi et al., 2014; Patil et al., 2016; Do et al., 2019). Despite the lack of research on the function of GmCHX20b, GmCHX20a and GmCHX1 may act together in a coordinated manner to treat both osmotic and ionic stress caused by high salinity (Jia et al., 2021). The ectopic expression of *GmCHX20a* led to salt hypersensitivity, which is consistent with increased Na^+^ uptake into the root. Meanwhile, the expression of *GmCHX1*, which facilitates Na^+^ exportation, is halted to avoid counteracting the function of GmCHX20a (Jia et al., 2021). In our study, *GmCHX20b* (*Glyma.13G131200*) was significantly downregulated in the leaves of the *gmsos1-1* mutant plants under salt stress. We speculate that *GmCHX20b* has a similar function as *GmCHX1* to reduce the accumulation of Na^+^ in the leaves. Na^+^/Ca^2+^ exchangers (NCXs) play an important role in Ca^2+^ homeostasis in excitable animal cells. The *NCX-like* gene in Arabidopsis (*AtNCL*) had Na^+^/Ca^2+^exchange activity and was involved in Ca^2+^ homeostasis under salt stress in *Arabidopsis*. The *atncl* mutants are salt-tolerant, while the *AtNCL* overexpression plants are salt-sensitive in Arabidopsis (Wang et al., 2012). In this study, *GmNCX1* (*Glyma.16G036000*) was significantly induced in the roots of the *gmsos1-1* plants under salt stress, while *GmNCX1* might function similarly as the *AtNCL*. Thus, the upregulation of *GmNCX1* may contribute to the hypersensitivity of the *gmsos1-1*. The SOS pathway is the best-characterized CBL-CIPK pathway. The salt-induced increase in cytosolic free Ca^2+^ is sensed by the calcium-binding protein AtSOS3/AtCBL4 which binds to the serine/threonine protein kinase AtSOS2/AtCIPK24 (Liu and Zhu, 1998; Halfter et al., 2000; Liu et al., 2000). The AtSOS2-AtSOS3 complex phosphorylates AtSOS1, transporting sodium out of the cell (Halfter et al., 2000). In this study, genes encoding CIPK9 (*Glyma.*16G020200), at least ten EF-hand calcium-binding domain-containing proteins, and other protein kinases are up or downregulated in the roots of the *gmsos1-1* mutant plants under salt stress (Supplementary Table 5), suggesting that malfunction of the calcium-binding proteins or calmodulin-like (CMLs) and CIPKs might contribute to the hypersensitive phenotype of the *gmsos1-1* mutant to salt stress.

In summary, dysfunction of the *GmSOS1* renders hypersensitivity to salt stress, which correlates with excessive Na^+^ accumulation and K^+^ efflux in the roots. This reverse genetic approach demonstrated that SOS1 function is conserved in eudicots (Arabidopsis sand soybean) and monocots (rice) of angiosperm species. The transcriptomic profiles of the roots of the *gmsos1-1* mutant plants provide evidence of the critical and global regulatory role of GmSOS1 in soybean salinity stress responses. Findings from this study provide strong genetic evidence for *GmSOS1* as a critical locus for future molecular breeding of salt-tolerant soybean cultivars. For example, as genome editing technologies develop, researchers could enhance the transcription of *GmSOS1* by altering its promoter (i.e., insertion of an enhancer element), deactivate its transcriptional repressors, or enhance the transporter activities of GmSOS1 by expressing a constitutively active form of GmSOS1.

## Accession Numbers

Sequence data from this article can be found in Phytozome (https://phytozome.jgi.doe.gov/pz/portal.html#) under accession numbers *AtSOS1* (AT2G01980.1) and *GmSOS1* (*Glyma.08G092000.1* and NP_001244939.1).

## Data Availability Statement

The datasets presented in this study can be found in online repositories. The names of the repository/repositories and accession number(s) can be found below: National Center for Biotechnology Information (NCBI) BioProject, accession no: PRJNA810576.

## Author Contributions

LX, JZ, and QZ supervised and designed the experiments. MZ, JC, LY, XL, YG, and FJ performed the experiments and analyzed the data. TZ performed the bioinformatics analysis. LX, SA, TX, and JZ wrote the manuscript. All authors reviewed and approved the final version of the manuscript.

## Conflict of Interest

The authors declare that the research was conducted in the absence of any commercial or financial relationships that could be construed as a potential conflict of interest.

## Publisher’s Note

All claims expressed in this article are solely those of the authors and do not necessarily represent those of their affiliated organizations, or those of the publisher, the editors and the reviewers. Any product that may be evaluated in this article, or claim that may be made by its manufacturer, is not guaranteed or endorsed by the publisher.

## Footnotes

1
youngerusa.com

2
http://www.ncbi.nlm.nih.gov

3
http://www.csbio.sjtu.edu.cn/bioinf/plant-multi/

4
https://services.healthtech.dtu.dk/service.php?TMHMM-2.0
