# Supplementary material for: A Putative Plasma Membrane Na+/H+ Antiporter GmSOS1 Is Critical for Salt Stress Tolerance in Glycine max
Source: Front Plant Sci. 2022 May 16;13:870695. doi: 10.3389/fpls.2022.870695 (PMC9149370; doi:10.3389/fpls.2022.870695)
Supplement: Supplementary file 1 [file Data_Sheet_1.PDF]

Supplementary Figure 1

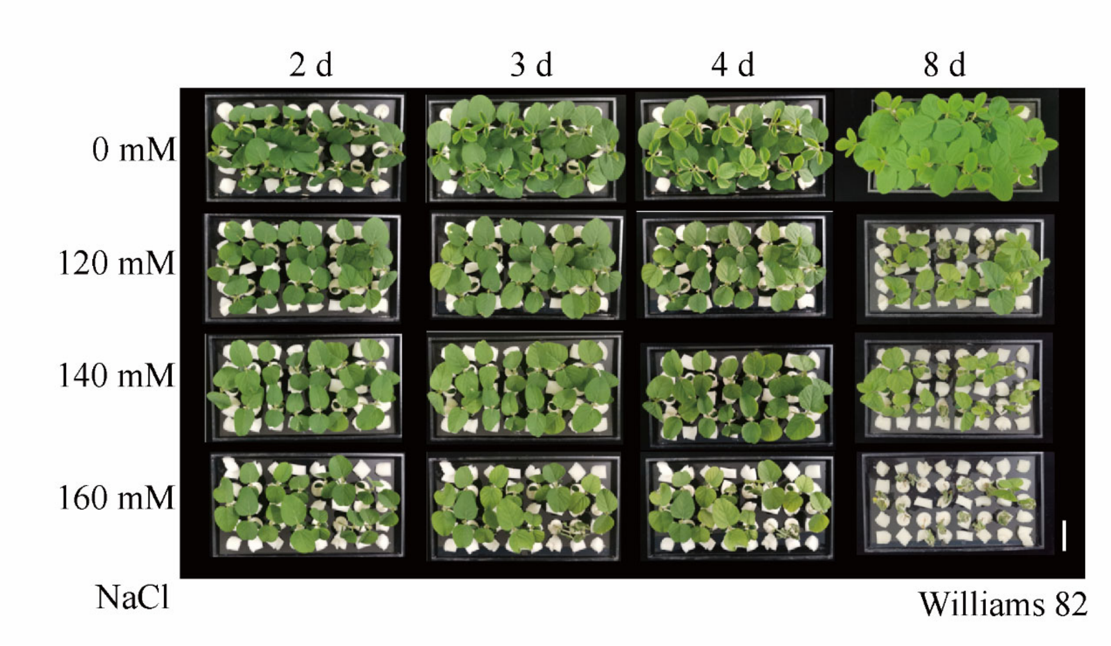

Figure S1. Salt sensitivity assay of soybean seedlings.
The Williams 82 soybean plants were cultivated in Hoagland medium containing various concentrations of NaCl from VC stage to V2 stage. Plants were photographed after 2, 3, 4 or 8 days of the treatment. Bar = 6 cm.

Supplementary Figure 2

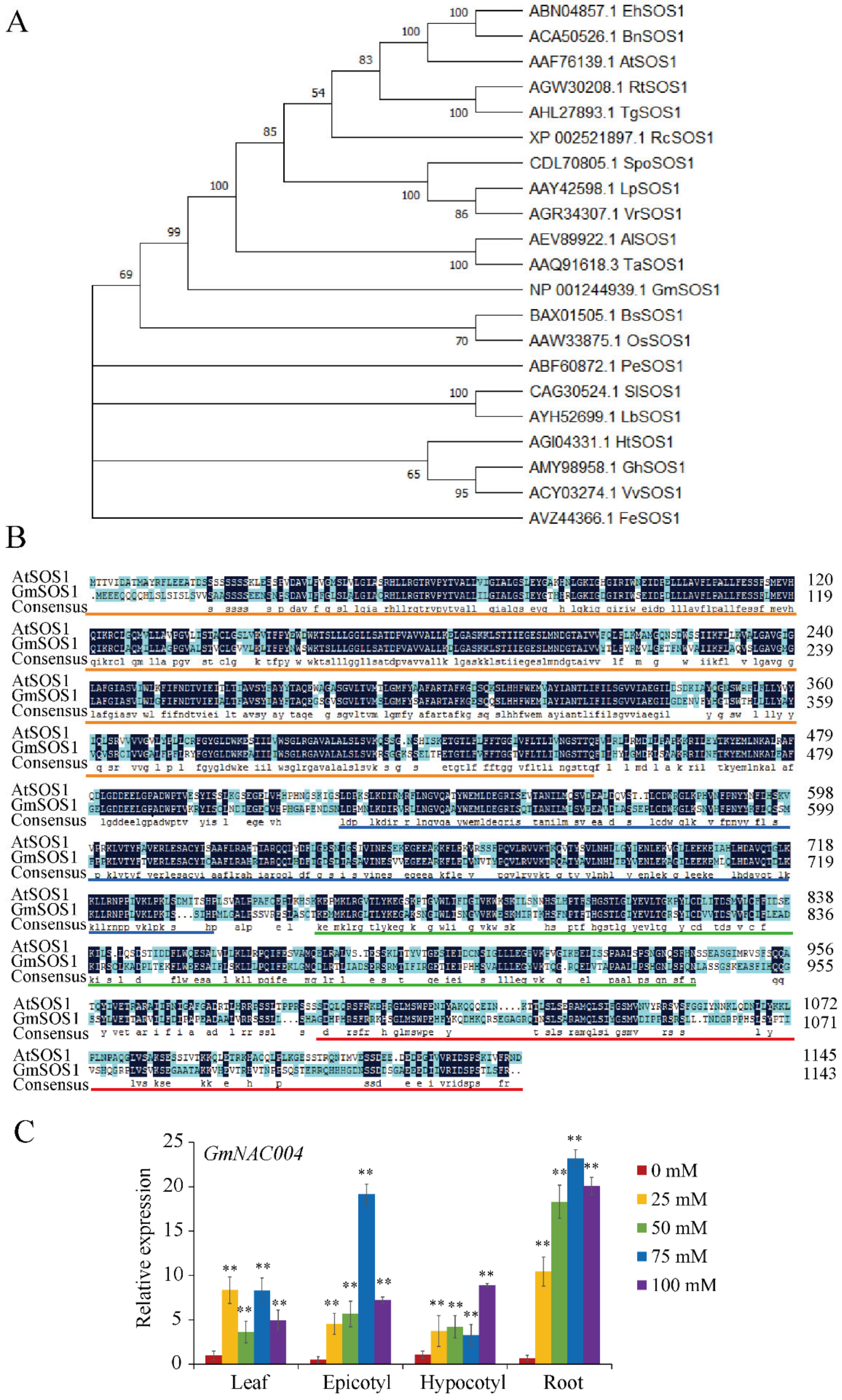

Figure S2. Comparison of GmSOS1 with its homologs in other species and expression pattern of *GmNAC004* in soybean.
(A) Phylogenetic analysis of GmSOS1 and its close homologs from other species by using the complete protein sequences. Bootstrap values (1000 replicates) are added to the phylogenetic tree.
(B) Alignment of the amino acid sequences of GmSOS1 and AtSOS1. Blue shading represents the conserved amino acids. Cyan shading represents the amino acids which are not conserved. Orange line, N-terminal transmembrane domain; blue line, Intracellular domain homologous to AtNHX8; green line, C-terminal cyclic nucleotide binding domain; red line, Self-inhibition domain**.**
(C) *GmNAC004* transcript levels in various tissues determined by qRT-PCR analysis. The RNA samples were extracted from unifoliate leaf, root, epicotyl and hypocotyl subjected to 0, 25, 50, 75, or 100 mM NaCl for 10 days. Values are means ± SD (n = 3). Significant differences in mean values relative to the mean value of unstressed plants in each tissue are indicated by Student's t-tests (** $p < 0.01$).

Supplementary Figure 3

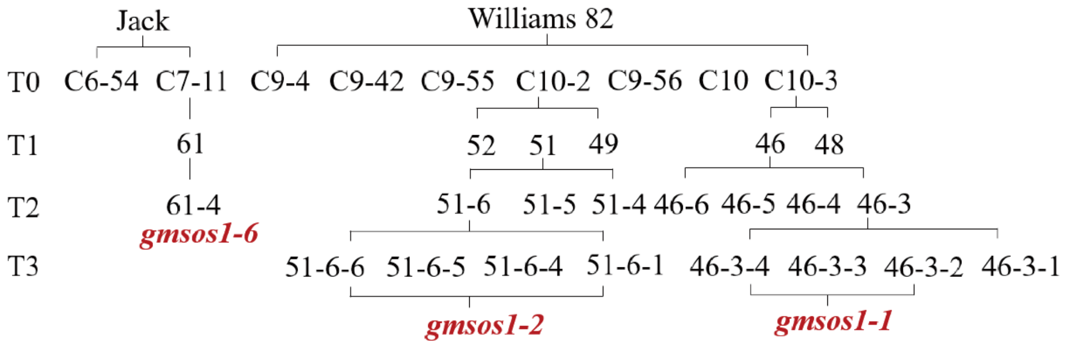

Figure S3. Identification of the *gmsos1* mutants.
The flowchart of the *gmsos1* mutants in T_0, T_1, T_2 and T_3 generations.
